# 

**Published:** 1855-07

**Authors:** 


JULY, 1855.

### THE Dental Register of the West.

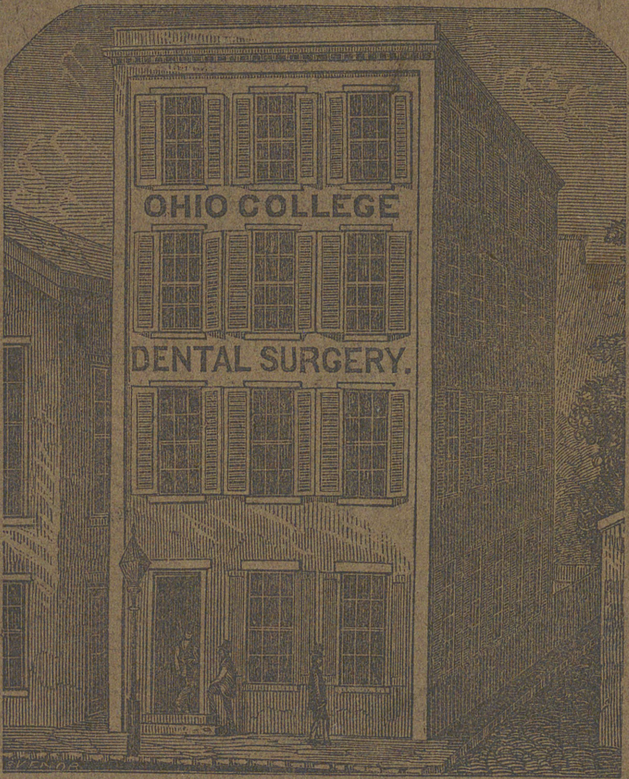

JAMES TAYLOR, M. D., D. D. S., Editor.

Published Quarterly at $2 per Annum.

CINCINNATI:
T. WRIGHTSON & CO., PRINTERS,
167 Walnut Street.

